# Baicalin Inhibits Airway Smooth Muscle Cells Proliferation through the RAS Signaling Pathway in Murine Asthmatic Airway Remodeling Model

**DOI:** 10.1155/2023/4144138

**Published:** 2023-02-13

**Authors:** Lingli Hu, Lulu Li, Chen Yan, Yuxue Cao, Xiaohong Duan, Jing Sun

**Affiliations:** ^1^Department of Integrative Medicine, Huashan Hospital, Fudan University, Shanghai 200040, China; ^2^Institute of Integrated Traditional Chinese and Western Medicine, Huashan Hospital, Fudan University, Shanghai 200040, China; ^3^Department of Oncology, Shanghai Municipal Hospital of Traditional Chinese Medicine, Shanghai University of Traditional Chinese Medicine, Shanghai 200040, China

## Abstract

**Background:**

Studies that looked at asthma airway remodeling pathogenesis and prevention have led to the discovery of the rat sarcoma viral oncogene (RAS) signaling pathway as a key mechanism that controls airway smooth muscle cell (ASMC) proliferation. Baicalin has great anti-inflammatory, proliferation-inhibited, and respiratory disease-relieving properties. However, the inhibitory effects and mechanisms of baicalin on ASMC-mediated airway remodeling in mice are still poorly understood.

**Methods:**

After establishing the asthmatic mice model by ovalbumin (OVA) and interfering with baicalin, airway remodeling characteristics such as airway resistance, mRNA, and protein expression levels of remodeling-related cytokines were measured by histopathological assessment, quantitative real-time polymerase chain reaction (qPCR), enzyme-linked immunosorbent assay (ELISA), and western blot. Further efforts on detailed mechanisms were used antibody arrays to compare the expression and activation of proteins involved in the RAS signaling pathway. In addition, validation experiments were performed in ASMC proliferation model and low-expression cells of the target gene by using shRNA.

**Results:**

In OVA-induced asthmatic mice model, baicalin significantly reduced the infiltration of inflammatory cells in lung tissue, attenuated airway resistance, and decreased mRNA and protein expression levels of remodeling-related cytokines such as interleukin-13 (IL-13), vascular endothelial growth factor (VEGF), transforming growth factor-beta 1 (TGF-*β*1), matrix metallopeptidase 9 (MMP9), and tissue inhibitor of metalloproteinase 1 (TIMP1). The results of antibody arrays involved in RAS signaling pathway revealed that OVA and baicalin administration altered the activation of protein kinase C alpha type (PKC-*α*), A-rapidly accelerated fibrosarcoma (A-RAF), mitogen-activated protein kinase 2 (MEK2), extracellular regulated MAP kinase (ERK), MAPK interacting serine/threonine kinase 1 (MNK1), and ETS transcription factor 1 (ELK1). The above results were further verified in the ASMC proliferation model. A-RAF silencing (shA-RAF) could promote ASMC proliferation and downregulate p-MEK2, p-ERK, p-MNK1, and p-ELK1 expression.

**Conclusion:**

The effects of baicalin against airway remodeling and ASMC proliferation might partially be achieved by suppressing the RAS signaling pathway. Baicalin may be a new therapeutic option for managing airway remodeling in asthma patients.

## 1. Introduction

Asthma is a common and frequently occurring disease that seriously affects human health [1]. The frustrating thing is that asthma patients with airway remodeling are less responsive to anti-inflammatory agents and bronchodilators, the standard of care for airway diseases [2]. Airway remodeling encompasses all structural alterations of the airways, including airway epithelial injury, subepithelial fibrosis and epithelial-mesenchymal transition (EMT), airway smooth muscle cell (ASMC) hyperplasia, goblet cell hyperplasia, and angiogenesis [3–5]. Therefore, clarifying the occurrence and development mechanisms and identifying effective interventions to prevent and cure airway remodeling are urgent measures for asthma.

Excessive proliferation of ASMCs is the main pathological feature of asthmatic airway remodeling. In asthma, the proliferation of ASMCs is inversely associated with lung function. Studies have shown that multiple factors promote the proliferation of ASMCs, resulting in airway wall thickening, lumen narrowing, and airway hyperresponsiveness (AHR) [6, 7]. Recent studies in ASMC proliferation have led to the discovery of that all extracellular signals that proliferate ASMCs converge on p21RAS [8]. RAS activation initiates ERK and phosphatidylinositol-3-kinase (PI3K); both ERK and PI3K upregulate CyclinD1 expression levels, accelerate the cell cycle, and promote cell proliferation. ERK signaling pathway is involved in hyperproliferation and differentiation of various types of cells [9]. Studies by Bai et al. have shown that the proliferation activity of ASMCs increases in rat asthma models, and the ERK signaling pathway was the principal character in the regulation of ASMC proliferation in asthma airway remodeling [10, 11]. Although previous studies have recognized the significance of RAS signaling in cell proliferation, none of the research has elucidated the regulatory mechanism and the effects of RAS signaling pathway on the proliferation of ASMCs.

Baicalin (PubChem CID: 64982), a flavonoid compound distilled from a traditional Chinese medicinal herb Skullcap (Huang-Qin, a medicinal plant), has been used as a therapeutic drug for various diseases for centuries because of its high medicinal value [12]. A comprehensive review of baicalin-related studies indicates that baicalin has great anti-inflammatory, proliferation-inhibited, and respiratory disease-relieving properties [13]. Studies have noticed that baicalin could ease asthma symptoms and prolong the incubation period of asthma by affecting the high mobility group box-1/Toll-like receptor 4 signaling pathway [14]. Previous trials have also shown that baicalin inhibits the proliferation of human glioblastoma cells through a Ca^2+^-dependent pathway [15]. Moreover, during oxidative stress regulation, PI3K/Akt/NRF2 are key factors associated with the healing effects of baicalin on ulcerative colitis and cholestasis [16]. However, the exact intervening role and underlying mechanism of baicalin in airway remodeling and even ASMC proliferation have not yet been clearly elucidated. Also, how does baicalin initiate intracellular signaling cascades is still unclear.

In this experiment, we intervened the mouse model of asthmatic airway remodeling by baicalin. We examined whether the improvement effect of baicalin on airway remodeling was achieved by modulating the activity of the RAS signaling pathway to inhibit the ASMC proliferation, providing new ideas and directions to explore the potential alternative therapy of asthma and the possible mechanism of baicalin to treat asthma, especially refractory asthma related to airway remodeling.

## 2. Materials and Methods

### 2.1. Animals

In this study, OVA- (Sigma-Aldrich, St. Louis, MO, USA) challenged mice were used as experimental models of asthma. The protocol was approved by the Institutional Animal Care and Use Committee of Fudan University (authorization number: 2019-10-HSYY-DJC-01). Female mice (BALB/c, Department of Laboratory Animal Science, School of Medicine, Fudan University) weighing 18–22 g (5 ± 1 weeks old) were purchased. Mice were housed in an environment where there were no specific pathogens, temperature and humidity were controlled, and water and food were given ad libitum.

### 2.2. Treatment of Mice

The protocol described in Supplementary Figure 1A was employed to induce airway remodeling. Mice were randomly divided into 6 groups of 10 mice each. Murine asthmatic models were established by chronic OVA sensitization and challenge as described previously with minor modifications [17]. Briefly, mice were multiply sensitized with 20 *μ*g OVA and 2 mg aluminum hydroxide (Thermo Scientific, Inc., Waltham, MA USA) in 0.2 mL of saline solution by i.p. injection on days 0, 7, 14, and 21 and challenged by exposure to 2% OVA solution (*w*/*v*) through an ultrasonic nebulizer for 30 min from day 25 to day 80. Mice in the normal control group were sensitized and challenged with saline. From day 25, mice in control and asthmatic model (OVA) groups were orally administrated with 200 *μ*L saline, and mice for baicalin treatment groups were orally administrated with 200 *μ*L different doses of baicalin (50, 100, and 200 mg/kg) (Shanghai Ronghe Corporation, Shanghai, China). For DEX treatment, mice in DEX groups were orally administrated with 200 *μ*L DEX-1 mg (Xinhua Pharmaceutical Co., Ltd., Shandong, China). Mice were euthanized by cervical dislocation after a lung function test on day 81. The blood (serum) was collected for cytokines determination, and lung tissues were collected for histopathological analysis and mRNA and proteins detection.

### 2.3. Pulmonary Function Test

For measuring the pulmonary function of asthmatic mice, the protocol described by Pichavant et al. [18] was employed. Mice are placed in the main chamber of the whole-body plethysmograph from Buxco (Buxco Electronics, Troy, NY, USA). The mice in the chamber were atomized inhalation with 3.125 mg/mL, 6.25 mg/mL, and 12.5 mg/mL methacholine (Mch) (Sigma-Aldrich; St. Louis, MO, USA) in turn. The atomization time was 10 seconds, and the recording time was 3 minutes, with an interval of 4 minutes. After being atomized by the atomizer, Mch entered the airway of mice with the airflow. The response of the airways to an inhaled stimulus MCh was measured and compared to the response to a control, normal saline. RL% = (RL value stimulated by Mch − RL value stimulated by normal saline)/RL value stimulated by normal saline × 100%. Following the respiratory function measurements, mice lungs were collected for analysis.

### 2.4. Histopathological Assessment

Mice lung sections (4 *μ*m) were stained with hematoxylin and eosin (H&E), periodic acid-Schiff (PAS), and Masson's Trichrome (Masson) staining. Then, a light microscope at high power (×100) in a blind fashion performed histopathological assessment in the bronchus. The specific method was consistent with our previous experiment [17]. The following indicators include bronchial basement membrane perimeter (Pbm), smooth muscle area (Wam), inner airway area (Wai), amount of ASMC nucleus (N), mucus staining area of the airway wall (Mus), and collagen around airway wall (Wcol) which should be measured by the Image-Pro Plus medical image analysis system. All the data measured was standardized using Pbm.

### 2.5. Antibody Array Analysis

We used snap-frozen lung samples of control, OVA, baicalin-treated, and DEX-treated mice to compare the expression and activation of proteins involved in the RAS signaling pathway. All samples were operated according to the Full Moon Company's standard operating procedures using its protein chip and related supporting kit. All samples were analyzed with phospho Exploerer (PEK208) by the H-Wayen Biotechnologies.

### 2.6. ASMC Culture and Treatment

The culture medium of ASMCs consisted of DMEM (Hyclone, South Logan, UT, USA) with 1% penicillin–streptomycin and 10% FBS. For the proliferation assay, ASMCs were cultured with 10 ng/mL PDGF (R&D Systems, Minneapolis, MN, USA) for 24 h to construct cell proliferation model. Baicalin (20 *μ*mmol/L, 40 *μ*mmol/L, and 60 *μ*mmol/L) and DEX (10 *μ*mmol/L) were treated 1 h before PDGF to interfere with the effect of PDGF. As baicalin and DEX were dissolved by dimethyl sulfoxide (DMSO) (Sigma-Aldrich, St. Louis, MO, USA), ASMCs were also treated with the same volume of vehicle (DMSO).

### 2.7. Proliferation and Viability Assays

Proliferation and viability assays of mouse ASMCs (Shanghai Yaji Biotechnology Co., Ltd., Shanghai, China) were performed with methylthiazolyldiphenyl-tetrazolium bromide (MTT) (Beyotime, Shanghai, China) and 5-ethynyl-2′-deoxyuridine (EdU) (Beyotime, Shanghai, China) measurement. ASMCs were cultured with PDGF or baicalin, and HEK293T cells were transfected with shA-RAF (Sangon Biotech Co., Ltd, Shanghai, China) or shNC (Sangon Biotech Co., Ltd, Shanghai, China) only and seeded in triplicate into 96-well plates at a density of 7 × 10^3^ cells/well. In addition, cells were cultured for 48 h, and 10 *μ*L of 5%MTT reagent was added to each well, followed by incubation for 4 h. Absorbance was measured at 570 nm with a microplate reader. For the viability assay, 2 × 10^5^ cells were added to the 24-well plates, and EdU (DAB) measurement was performed at 48 h. All the experiments were repeated three times.

### 2.8. A-RAF Low Expression and Transfection with A-RAF shRNA

HEK293T cells were independently transfected with pSGU6-A-RAF-shRNA-RFP in the presence of Lipofectamine 3000 (Invitrogen, San Diego, USA). shRNA negative control (shRNA-NC, shNC) vector was used as control. The A-RAF shRNA and shNC were supported by Sangon Biotech Co., Ltd. (Shanghai, China). HEK293T cells were transfected according to the manufacturer's instructions with 2 *μ*g of shRNA only to decrease A-RAF expression. The A-RAF expression in HEK293T cell was detected by qPCR and western blot.

### 2.9. Western Blot

Protein extraction, concentration determination, and the following process were consistent with our previous experiment [19]. All antibodies were purchased from Cell Signaling (Houston, TX, USA). The dilution ratio of antibodies used in this research was as follows: anti-VIMENTIN (1 : 2000 dilution), anti-*α*-SMA (1 : 3000 dilution), anti-GAPDH (1 : 5000 dilution), anti-TGF-*β*1 (1 : 1000 dilution), anti-MMP9 (1 : 1000 dilution), anti-PKC-*α* (1 : 1000 dilution), anti-p-PKC-*α* (1 : 1000 dilution), anti-A-RAF (1 : 1000 dilution), anti-p-A-RAF (1 : 1000 dilution), anti-MEK2 (1 : 1000 dilution), anti-p-MEK2 (1 : 1000 dilution), anti-ERK (1 : 1000 dilution), anti-p-ERK (1 : 1000 dilution), anti-MNK1 (1 : 1000 dilution), anti-p-MNK1 (1 : 1000 dilution), anti-ELK1 (1 : 1000 dilution), anti-p-ELK1 (1 : 500 dilution), and HRP-labeled secondary antibody (1 : 5000 dilution).

### 2.10. ELISA Analysis

According to the manual, appropriate antibody ELISA kits (BioTNT, Co., Ltd, Shanghai, China) were used to detect the protein expression of markers related to airway remodeling in the serum.

### 2.11. Quantitative Real-Time Polymerase Chain Reaction (qPCR)

The process detects mRNA expression by qPCR agrees with our earlier experiments [19]. Total RNA was extracted by using TRIzol™ reagent (Takara Bio, Kusatsu, Shiga, Japan) following the manufacturer's protocol. Total RNA was reverse transcribed into cDNA using the iScriptDNA Synthesis kit (Bio-Rad Laboratories, California, USA) using the following temperature protocol: priming at 25°C for 5 min, RT at 46°C for 20 min, reverse transcriptase inactivation at 95°C for 1 min, and then holding at 4°C. cDNA was subjected to qPCR using Power SYBR Green PCR Master Mix (Applied Biosystems, Thermo Fisher Scientific, Inc., Waltham, MA, USA) and the ABI 6500 fast Real-Time PCR system (Applied Biosystems; Thermo Fisher Scientific, Inc., Waltham, MA, USA). The thermocycling conditions of the qPCR steps were as follows: activation (temperature: 50°C; duration: 2 min; cycles: hold), Dual-LockTM DNA polymerase (temperature: 95°C; duration: 2 min; cycles: hold), denaturation (temperature: 95°C; duration: 15 sec; cycles: 40), and annealing/extension (temperature: 60°C; duration: 1 min; cycles: 40). The specific primers (Sangon Biotech Co., Ltd, Shanghai, China) were listed. Mus- *Gapdh*: AGGTCATCCCAGAGCTGAACG (forward), CACCCTGTTGCTGTAGCCGTAT (reverse); Mus-*α-Sma*: GTACCACCATGTACCCAGGC (forward), GCTGGAAGGTAGACAGCGAA (reverse); Mus-*Tgf-β1*: CTGCTGACCCCCACTGATAC (forward), GTGAGCGCTGAATCGAAAGC (reverse); Mus-*Mmp9*: CGCTCATGTACCCGCTGTAT (forward), CCGTGGGAGGTATAGTGGGA (reverse); Mus-*Vegf*: ACTGGACCCTGGCTTTACTG (forward), GCTTCGCTGGTAGACATCCA (reverse); Mus-*Timp1*: CAGATACCATGATGGCCCCC (forward), ACCGGATATCTGCGGCATTT (reverse); Homo-*GAPDH*: AGAAGGCTGGGGCTCATTTG (forward), AGGGGCCATCCACAGTCTTC (reverse); and Homo-*A-RAF*: ACCAACCGCCAACAGTTCTA (forward), CTACTACCGGCAGCATCAGT (reverse).

### 2.12. Statistical Analysis

Data were presented as mean ± SEM. An unpaired Student's *t*-test and an ANOVA with a Bonferroni post hoc test were used for single and multiple comparisons between two or more groups, respectively. *P* < 0.05 was considered statistically significant.

## 3. Results

### 3.1. Baicalin-Treated Mice Have Milder Airway Remodeling Characteristics

**Figure 1:**
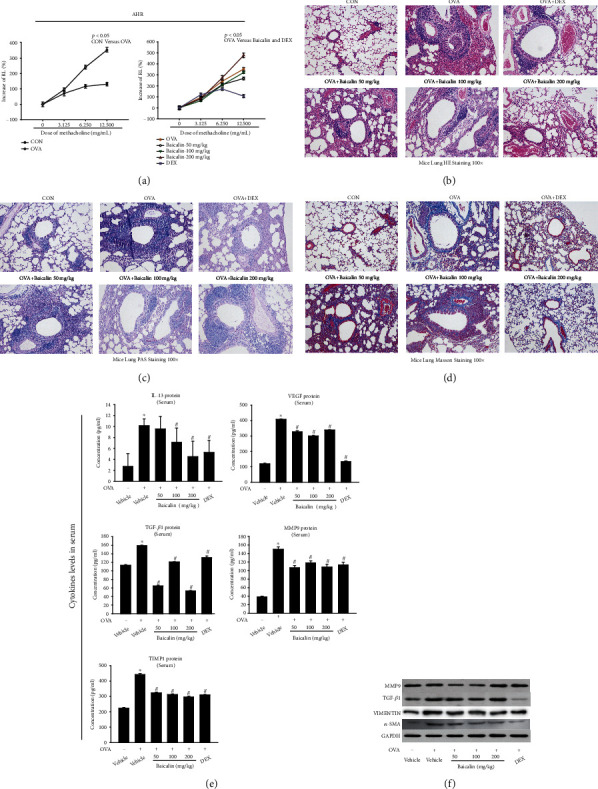
Effects of baicalin on AHR, inflammatory cell infiltration, lung histology, and airway remodeling. (a) RL was measured after treatment with methacholine. (b) HE staining was used to measure airway wall thickness, bronchial lumen, mucosal edema, and inflammatory cell infiltration. (c) PAS staining was used to measure mucus production. (d) Masson staining was used to measure collagen deposition around the airways (×100 magnification). (e) Protein expression of IL-13, VEGF, TGF-*β*1, MMP9, and TIMP1in serum was measured by ELISA. (f) Protein expression of *α*-SMA, VIMENTIN, MMP9, and TGF-*β*1 in lung tissue was measured by western blot. All data were shown as mean ± SEM. Sections are representative of *n* ≥ 6 mice from each group. ^∗^*P* < 0.05 versus vehicle. ^#^*P* < 0.05 versus OVA.

To investigate the effect of baicalin on OVA-induced asthmatic airway remodeling, we treated asthmatic airway remodeling mice with different strategies (Supplementary figure 1A), then assessed the resistance of lung (RL) by pulmonary function measurement, asthma inflammation, and remodeling by pathological assessment, western blot, and ELISA. RL is an indicator of bronchoconstriction, reflecting AHR, measured at baseline and after continuous administration of acetylcholine concentrations (3.125-12.5 mg/mL) [20]. As shown in Figure 1(a), baicalin (50 mg/kg and 100 mg/kg) decreased the RL that OVA upregulated. Asthma is characterized by a marked increase in inflammatory cell infiltration into the airways and the occurrence of airway remodeling. Compared with the CON group, lungs in OVA group had obvious inflammatory cell infiltration, mucosal edema, thickening of the airway wall, stenosis of the lumen, and increased secretions in the lumen, which was a typical feature that was significantly reduced by baicalin (Figure 1(b)). In addition, remodeling indicators Wam/Pbm (represents the thickness of smooth muscle layer), Wai/Pbm (represents the thickness of bronchial inner wall), and N (represents the number of smooth muscle nuclei) were recognized by the Image-Pro Plus software. These indicators were significantly increased in the OVA group while all decreased in baicalin (100 mg/kg) or DEX pretreatment group (Supplementary Figure. 1B-1D). The results of HE staining indicated that baicalin could significantly reduce the inflammation and airway wall thickness of asthmatic airway remodeling mice. Besides, PAS staining showed that PAS-positive cells in airway epithelium of baicalin group, number of goblet cells, and amount of mucus secretion were considerably lower than that of OVA group (Figure 1(c)). Muc/Pbm, an index reflecting mucus secretion, was significantly decreased in baicalin- (50 mg/kg and 200 mg/kg) treated group compared with that in the OVA group (Supplementary figure 1E). Masson's trichrome staining was used to indicate the collagen deposition around the airway of mice, in which the green part represented the collagen fiber deposition. As shown in Figure 1(d), compared with CON group, the collagen deposition in lung tissue, the area of collagen, and the thickness of trachea in OVA group increased. All the above pathological phenomena were relieved considerably in baicalin-treated mice. Wcol/Pbm was an index reflecting collagen deposition, which was also significantly decreased in baicalin- (50 mg/kg and 100 mg/kg) treated group compared with that in the OVA group (Supplementary figure 1F).

As shown in Figure 1(e), the levels of airway remodeling-related markers such as IL-13, VEGF, TGF-*β*1, MMP9, and TIMP1 in the OVA group increased importantly. However, these were all suppressed by baicalin (100 mg/kg and 200 mg/kg), and VEGF, TGF-*β*1, MMP9, and TIMP1 could also be suppressed by baicalin (50 mg/kg). Furthermore, we also detected the protein expression of *α*-SMA, VIMENTIN, MMP9, and TGF-*β*1 in lung tissue and found that their levels were all decreased in baicalin (100 mg/kg) group compared with OVA group (Figure 1(f) and Supplementary figure 1G).

Taken together, these results demonstrated that baicalin could reduce OVA-induced asthmatic remodeling by reducing AHR, pulmonary inflammation, goblet cell hyperplasia, collagen deposition, and remodeling-related cytokine expression. Furthermore, through the comprehensive judgment of baicalin on the phenotypic improvement of asthma airway remodeling, we believe that baicalin 100 mg/kg may be a better dose based on the above results.

### 3.2. Baicalin Decreases RAS Signaling Pathway Activation

We used antibody arrays to compare the expression and activation of proteins involved in the RAS signaling pathway to understand the mechanism of baicalin-driven asthma and airway remodeling relief. To this end, we compared snap-frozen lung samples of CON, OVA, baicalin 100 mg/kg, and DEX group to observe the changes in protein phosphorylation among groups. OVA and baicalin administration altered large amounts of signaling molecules (Supplementary figure 2). Among them, the proteins that OVA and baicalin oppositely regulated by each other were PKC-*α*, A-RAF, MEK2, ERK, MNK1, and ELK1.

**Figure 2:**
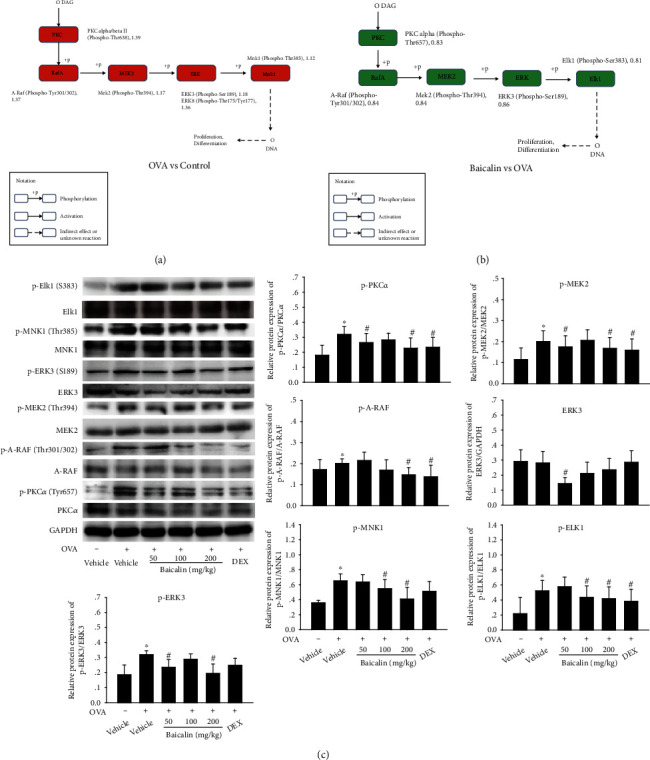
Effects of baicalin on RAS signaling pathway. (a, b) The key branching pathway was composed of the differential proteins screened by antibody array in CON and OVA groups and OVA and baicalin groups. (c) The protein band diagram and quantitative statistical diagram of diﬀerential proteins of RAS signaling pathway in lung tissue. All data are shown as mean ± SEM. The data are representative of *n* ≥ 6 from each group. ^∗^*P*< 0:05 versus vehicle. ^#^*P*< 0:05 versus OVA.

Compared with CON group, the differentially expressed proteins of RAS signaling pathway in OVA group were PKC-*α*, A-RAF, MEK2, ERK, and MNK1 (Figure 2(a)). The above proteins harbored the ability to regulate cell proliferation and showed an upward expression trend, indicating that OVA sensitization and aerosol inhalation could promote cell proliferation and airway reconstruction in mice. Compared with the OVA group, the differentially expressed protein of RAS signal pathway in baicalin group were PKC-*α*, A-RAF, MEK2, ERK, and ELK1 (Figure 2(b)). The above proteins also can regulate cell proliferation but show a downward expression trend, suggesting that baicalin could effectively interfere with asthmatic airway reconstruction by inhibiting cell proliferation.

Western blot analysis revealed that the activity of differential proteins was increased in OVA samples, while the levels of differential protein were not significantly affected by OVA. On the contrary, the activity of differential proteins was decreased in baicalin samples, while the expression levels remained unchanged (Figure 2(c)). The above results suggested that baicalin could inhibit the RAS signaling pathway activated by OVA, consistent with the microarray results. It further indicated that baicalin may intervene airway remodeling in asthma by activating RAS signaling pathway.

### 3.3. Baicalin Inhibits RAS Signaling Pathway Activation in ASMC Proliferation Model

**Figure 3:**
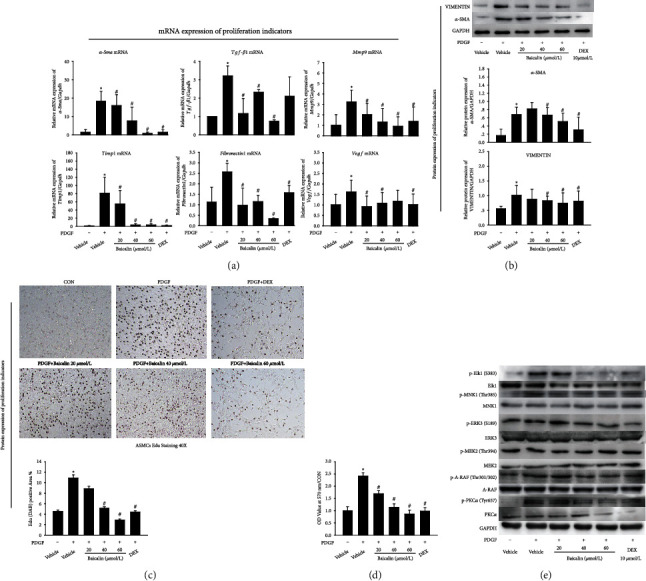
Baicalin inhibits ASMC proliferation and RAS signaling pathway activation. (a) mRNA expression of *α-Sma/Tgf-β1/Mmp9/Timp1/Fibronectin1* and *Vegf* was measured by qPCR. (b) Protein expression of *α*-SMA and VIMENTIN was measured by western blot. The relative density of each protein was calculated. (c) ASMC proliferation was detected by the EdU (DAB) test. (d) MTT assay was used to assess ASMC proliferation. (e) The expression of differential proteins in key branching pathway of ASMCs was detected by western blot. All data were shown as mean ± SEM. The data are representative of *n* ≥ 6 from each group. ^∗^*P* < 0.05 versus vehicle. ^#^*P* < 0.05 versus PDGF.

To investigate whether baicalin affects ASMC proliferation via the RAS signaling pathway, we first induced an ASMC proliferation model, treated it with baicalin, and detected proliferation indicators by qPCR/western blot/EdU (DAB) and MTT. qPCR results disclosed that PDGF increased mRNA expression of proliferation markers such as *α-Sma/Tgf-β1/Mmp9/Timp1/Fibronectin1* and *Vegf* (Figure 3(a)). Meanwhile, the western blot data for evaluating the expression of proliferation markers were consistent with the results observed by qPCR (Figure 3(b)). And baicalin had potently decreased the mRNA and protein expression of *α*-SMA/TGF-*β*1/MMP9/TIMP1/FN1 and VEGF. The above results demonstrated that the proliferation model of ASMCs was successfully constructed, and baicalin could inhibit the proliferation of ASMCs.

Further, the EdU (DAB) test was also used to detect the effect of baicalin on ASMC proliferation. The cell proliferation was evaluated according to the size and depth of the brown area after DAB staining. The larger the brown area and the darker the color, the faster the cells proliferate. As shown in Figure 3(c), the brown area of cells in PDGF group was more extensive and darker, indicating that ASMCs proliferated faster. In contrast, the cell proliferation of baicalin and DEX group was slower. MTT assay also showed that the absorbance of PDGF group 4at 570 nm was higher than that of the CON group. Baicalin and DEX intervention could reduce the absorbance value (Figure 3(d)), suggesting that baicalin effectively inhibited cell proliferation. The above results may indicate that baicalin can inhibit the proliferation of ASMCs.

**Figure 4:**
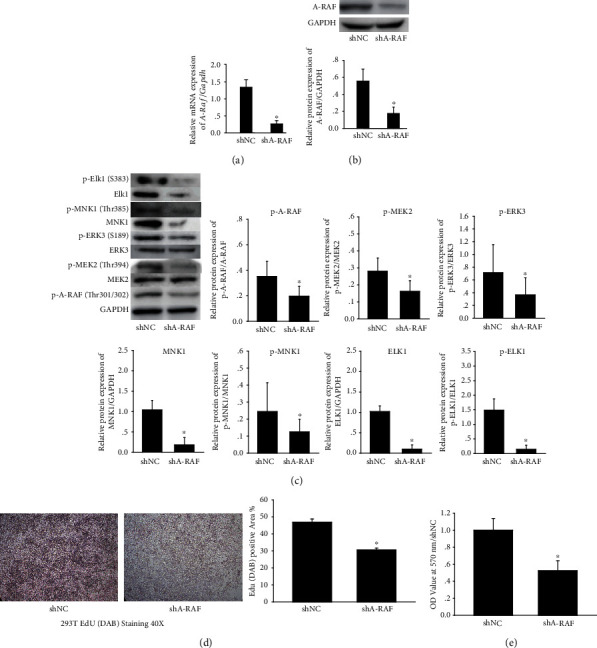
Effects of A-RAF shRNA on RAS signaling pathway and cell proliferation. (a) The mRNA expression of A-RAF was measured by qPCR in HEK293T cell after A-RAF ablation. (b) The protein expression of A-RAF was measured by western blot in HEK293T cell after A-RAF ablation. (c) The expression of differential proteins in key branching pathway of HEK293T cell was detected by western blot after A-RAF ablation. The relative density of each protein was calculated. (d) The proliferation of HEK293T cell was detected by the EdU (DAB) test. (e) The proliferation of HEK293T cell was detected by MTT assay. All data were shown as mean ± SEM. All the data are representative of *n* ≥ 6 from each group. ^∗^*P* < 0.05 versus shNC.

To study whether baicalin affects ASMC proliferation via RAS signaling pathway, we then detected the expression and activity of differential proteins in ASMC proliferation model by western blot. As shown in Figure 4(e) and Supplementary figure 3, baicalin decreased the expression of the phosphorylated differential protein upregulated by OVA, suggesting that baicalin inhibited the activity of key branching proteins activated by OVA. The above results indicated that baicalin could inhibit RAS signaling pathway activation, which was consistent with the mice lung and protein chip detection. It further showed that baicalin inhibited the RAS pathway in the ASMC proliferation model.

### 3.4. A-RAF Ablation Prevents RAS Signaling Pathway Activation and Inhibits Cell Proliferation

Our previous results revealed that baicalin prevented the RAS signaling pathway in the ASMC proliferation model. To identify the role of the RAS pathway in cell proliferation, a RAS signaling pathway blocked cell model was established based on the low expression of A-RAF by its specific shRNA. Then A-RAF mRNA and protein were detected to confirm whether the establishment of a low-expression cell line was successful or not. Results have proven that the A-RAF mRNA and total protein expression were much lower than that in the shNC group (Figures 4(a) and 4(b)).

Subsequently, we detected the expression of A-RAF downstream proteins in the RAS signaling pathway; results showed that the expression of total proteins and phosphorylated proteins of MEK2/ERK/ELK1 and MNK1 was reduced following A-RAF knockdown (Figure 4(c)).

The EdU (DAB) and MTT experiments were used to examine the cell proliferation of shNC and shA-RAF groups. Results revealed that the cell proliferation of shA-RAF group was slower than that of the shNC group (Figures 4(d) and 4(e)), indicating that knocking down A-RAF and blocking the RAS signaling pathway could effectively slow down cell proliferation.

## 4. Discussion

Baicalin is the main active component of *Scutellaria baicalensis* [21], which exhibits a wide range of biological activities in vivo, including anti-inflammatory, antibacterial, antiviral, and antioxidant [22–25]. However, the mechanisms of baicalin in bronchial asthma and airway remodeling remain ill-defined. In the present study, we revealed the role of baicalin in alleviating bronchial asthma and airway remodeling in vivo and in vitro [26]. As sex hormones are important in asthma pathogenesis, using female mice to construct asthma models could obtain more typical symptoms [27]. We used a female mouse model of asthmatic airway remodeling induced by OVA and found that baicalin significantly alleviated airway remodeling, including infiltration of inflammatory cells, airway mucus production, goblet cell proliferation, and AHR. Also, baicalin significantly attenuated the mRNA and protein expression levels of IL-13, VEGF, TGF-*β*1, MMP9, TIMP1, *α*-SMA, and VIMENTIN in the lung and serum. These antiairway remodeling effects of baicalin are supported by our previous study, in which baicalin successfully suppressed the release of inflammatory factors such as ET-1-, TGF-*β1*-, and IL-13-attenuated mucus cell hypertrophy and airway luminal narrowing in mice asthmatic model [26]. Baicalin ameliorates the pathogenesis and development of pediatric asthma by upregulating microRNA-103 and mediating the TLR4/NF-*κ*b pathway [28]. Therefore, baicalin may be a potential therapy for OVA-induced asthmatic airway remodeling.

The proliferation and migration of ASMCs are the key factors that induce airway remodeling in asthma. As ASMC proliferation continues to be intensively studied, although different factors regulate ASMC proliferation by various mechanisms, all extracellular signals that proliferate ASMCs converge on p21RAS [8]. Therefore, we used the RAS pathway phosphorylation antibody array to perform proteomic analysis on lung samples of the CON, OVA, baicalin 100 mg/kg, and DEX groups to obtain the phosphorylation site proteins that differ among groups and obtain key branching proteins of the RAS signaling pathway: PKC-*α*, A-RAF, MEK2, ERK, MNK1, and ELK1 (p-PKC*α*-Thr638, p-A-RAF-Tyr301/302, p-MEK2-Tyr394, p-ERK3-Ser189, p-ELK1-Ser383, and p-MNK1-Thr385). Western blot verification of differential proteins in lung indicated that OVA-induced upregulated phosphorylated differential proteins could be reversed by baicalin. It was worth noting that the intervention effect of the baicalin 100 mg/kg on key branching proteins was not the best in the western blot results. This contradictory result may be derived from the different levels of observed indicators, and the optimal dose of baicalin to intervene in asthmatic airway remodeling remained to be determined by further in-depth studies.

PKC-*α* is widely expressed in many tissues and is involved in cellular processes such as cell proliferation, differentiation, apoptosis, adhesion, and movement [29, 30]. PKC-*α* has previously been implicated in regulating proliferation through upregulating p21cip1in human glioma cells [31]. PKC-*α* can phosphorylate and activate A-RAF, thereby activating ERK-MAPK to promote cell growth and proliferation. The first discovered family member of the RAF family of serine/threonine kinases, A-RAF, is involved in critical cellular processes, including proliferation, differentiation, and survival. Activation of A-RAF by the phosphorylation at Ser 338 and Tyr341 is a crucial step in the above process [32, 33]. Studies have shown that the MAP kinase cascade involving A-RAF and MEK plays a prominent role in promoting ASMCs and skeletal muscle cell proliferation [34, 35]. ELK-1 is a transcription factor involved in ERK-induced cell proliferation, regulating cell growth, differentiation, and survival [36]. Research has pointed out that the ELK1-activated GPC3-AS1/GPC3 axis promotes the proliferation and migration of cervical cancer cells [37]. MAP kinase-interacting kinase 1 (MNK1) functions downstream of MAP kinases, such as p38 and ERK, promote proliferation and invasion of hepatocellular carcinoma [38]. Phosphorylation of eIF4E by MNKs supports protein synthesis, cell cycle progression, and proliferation in prostate cancer cells [39].

All these findings have demonstrated that the effector proteins screened out by protein chip are related to cell proliferation. Next, baicalin was used to intervene in the ASMC proliferation model and detect effector proteins. The results showed that baicalin had an inhibitory effect on the proliferation of ASMCs, which was consistent with some previous research. The research by Yang et al. has indicated that baicalin could suppress PDGF-induced rat ASMCs migration via the MAPK signaling pathway [40]. Moreover, baicalin regulates the development of pediatric asthma by inhibiting the abnormal proliferation of smooth muscle cells induced by PDGF-BB [28]. Our findings also suggested that baicalin had an inhibitory effect on the phosphorylation site proteins (p-PKC*α*-Thr638, p-A-Raf-Tyr301/302, MEK2-Tyr394, p-ERK3-Ser189, p-ELK1-Ser383, and p-MNK1-Thr385), which were consistent with the results of in vivo verification and antibody array.

Previous studies have shown that baicalin induces continuous activation of extracellular regulated protein kinase (ERK) 12 and protein kinase C (PKC) in a mouse model of acetaminophen-induced hepatotoxicity [41]. In HCT116 colon cancer cells, baicalin upregulated progesterone-induced decidual protein expression and activated RAS/RAF/MEK/ERK and p16INK4A/Rb signaling pathways [42]. This discrepancy may be due to differences in the tissues and mouse models. It is worth noting that the effector protein A-RAF screened by the antibody array was phosphorylated at Tyr301/302. In addition, both in vivo and in vitro verification results in this study showed that the phosphorylation activation site of A-RAF was Tyr301/302. Our findings were inconsistent with previous research; phosphorylation activation sites of A-RAF were Ser 338 and Tyr341. However, the mechanism of A-RAF complex activation is still not fully understood and requires further investigation [43, 44]. Researchers found that the RAF protein mutated from Tyr341 to Asp remained activated after membrane binding [45]. Therefore, there are still many other mechanisms for RAF activation, and Tyr301/302 screened and verified in this study may be another mechanism of RAF activation in asthma airway remodeling and ASMC proliferation.

To further demonstrate the role of key branching pathway in airway remodeling and cell proliferation, we constructed a cell model with low expression of effector proteins on this pathway by using shRNA technology. Western blot results indicated that blocking the key signaling pathway could inhibit the expression and activation of A-RAF downstream proteins. EdU (DAB) and MTT assay demonstrated that the cell proliferation rate of shA-RAF group was significantly lower than that of the shNC group, suggesting that the key branching pathway of RAS plays an important role in cell proliferation. The response of cells to growth signals mediated by Ras pathway has been widely studied in tumor diseases. The activation and mutation of Ras and B-Raf genes have been reported in various neoplastic diseases [46]. However, the RAS pathway, especially A-RAF, was rarely studied in the pathological process of other cell dysproliferative disorders. In this study, we screened the key RAS branching pathway starting from A-RAF by antibody array assays, and verified the role of this key branching pathway in airway remodeling and the regulatory effect of baicalin on it. The above findings were conducive to studying the biological effects of RAS signaling pathway, the impact of dysregulation/blocking RAS signaling pathway, and the development of small molecule inhibitors of this pathway. However, there are many limitations that need to be considered. Firstly, our study has not explored the in-depth mechanisms of baicalin modulating the RAS signaling pathway. Studies have shown that after being activated by many growth factors (such as EGF, FGF, and PDGF), receptor tyrosine kinases bind to adaptor protein ligands, promote the release of GDP and finally activate RAS. Therefore, more studies are needed to investigate the above RAS activation processes to seek possible mechanisms through which baicalin inhibits RAS signaling. Secondly, the results of our current work showed that the inhibition of the RAS signaling pathway might be one of the mechanisms that regulated the proliferation of ASMCs by baicalin. In fact, there are still many other signaling pathways left to be explored.

## 5. Conclusions

Our study evaluated the effect of baicalin on asthmatic airway remodeling via inhibiting ASMC proliferation by mediating the RAS signaling pathway. The present study suggested that the RAS signaling pathway can be used as a therapeutic drug target and pathway to alleviate airway remodeling. The application of baicalin and Traditional Chinese Medicines/formulas rich in baicalin may provide new ideas for the intervention of airway remodeling-related pulmonary diseases such as asthma. Additionally, baicalin could be used as a replacement/substitute treatment for refractory asthma that is resistant to asthma treatment medicines, especially glucocorticoids.

## Data Availability

The authors declare that the data presented in this study will be presented upon request from the corresponding author.

## Abbreviations

*α*-SMA: Alpha-smooth muscle actin
AHR: Airway hyperresponsiveness
ASMCs: Airway smooth muscle cells
DEX: Dexamethasone
DMSO: Dimethyl sulfoxide
EdU: 5-Ethynyl-2′-deoxyuridine
ELISA: Enzyme-linked immunosorbent assay
ELK1: ETS transcription factor 1
ERK: Extracellular regulated MAP kinase
H&E: Hematoxylin and eosin
HEK293T: Human embryonic kidney 293T cells
IL-13: Interleukin-13
Masson: Masson's trichrome
MEK2: Mitogen-activated protein kinase 2
MMP9: Matrix metallopeptidase 9
MNK1: MAPK interacting serine/threonine kinase 1
MTT: Methylthiazolyldiphenyl-tetrazolium bromide
OVA: Ovalbumin
PAS: Periodic acid-Schiff
PDGF: Platelet-derived growth factor
PI3K: Phosphatidylinositol-3-kinase
PKC-*α*: Protein kinase C alpha type (PKC-*α*)
qPCR: Quantitative real-time polymerase chain reaction
RAF: Rapidly accelerated fibrosarcoma
RAS: Rat sarcoma viral oncogene
RL: Resistance of lung
TGF-*β*1: Transforming growth factor-beta 1
TIMP1: Tissue inhibitor of metalloproteinase 1
VEGF: Vascular endothelial growth factor.

## Supplementary Materials

Supplementary Materials Figure S1: effects of baicalin on airway remodeling indicators in mice. (A) Schematic diagram of the experimental protocol. (B) Airway remodeling-related indicators Wam/Pbm (B), Wai/Pbm (C), and N/Pbm (D) were measured from H&E staining. (E) Muc/Pbm was measured from PAS staining. (F) Wcol/Pbm was measured from Masson staining. (G) Relative density of *α*-SMA, VIMENTIN, MMP9, and TGF-*β*1in the lung. All data were shown as mean ± SEM. The data are representative of *n* ≥ 5 from each group. ^∗^*P* < 0.05 versus vehicle. ^#^*P* < 0.05 versus OVA. Figure S2: differentially expressed proteins were screened out by an antibody array. (A) Differentially expressed proteins on the RAS signaling pathway between the CON and OVA groups. (B) Differentially expressed proteins on the RAS signaling pathway between the OVA and baicalin groups. All data were shown as mean ± SEM. The data are representative of *n* = 5 mice from each group. Figure S3: baicalin inhibits the RAS signaling pathway activation. (A) p-PKC*α*, (B) p-A-RAF, (C) p-ERK3, (D) p-MNK1, (E) ELK1, and (F) p-ELK1 protein expression in ASMCs. All data were shown as mean ± SEM. The data are representative of *n* ≥ 6 from each group. ^∗^*P* < 0.05 versus vehicle. ^#^*P* < 0.05 versus PDGF.
