# Supplementary material for: Baicalin Inhibits Airway Smooth Muscle Cells Proliferation through the RAS Signaling Pathway in Murine Asthmatic Airway Remodeling Model
Source: Oxid Med Cell Longev. 2023 Feb 13;2023:4144138. doi: 10.1155/2023/4144138 (PMC9940961; doi:10.1155/2023/4144138)
Supplement: Supplementary Materials — Figure S1: effects of baicalin on airway remodeling indicators in mice. (A) Schematic diagram of the experimental protocol. (B) Airway remodeling-related indicators Wam/Pbm (B), Wai/Pbm (C), and N/Pbm (D) were measured from H&E staining. (E) Muc/Pbm was measured from PAS staining. (F) Wcol/Pbm was measured from Masson staining. (G) Relative density of α-SMA, VIMENTIN, MMP9, and TGF-β1in the lung. All data were shown as mean ± SEM. The data are representative of n ≥ 5 from each group. ∗P < 0.05 versus vehicle. #P < 0.05 versus OVA. Figure S2: differentially expressed proteins were screened out by an antibody array. (A) Differentially expressed proteins on the RAS signaling pathway between the CON and OVA groups. (B) Differentially expressed proteins on the RAS signaling pathway between the OVA and baicalin groups. All data were shown as mean ± SEM. The data are representative of n = 5 mice from each group. Figure S3: baicalin inhibits the RAS signaling pathway activation. (A) p-PKCα, (B) p-A-RAF, (C) p-ERK3, (D) p-MNK1, (E) ELK1, and (F) p-ELK1 protein expression in ASMCs. All data were shown as mean ± SEM. The data are representative of n ≥ 6 from each group. ∗P < 0.05 versus vehicle. #P < 0.05 versus PDGF. [file 4144138.f1.doc]

## Supplementary Materials


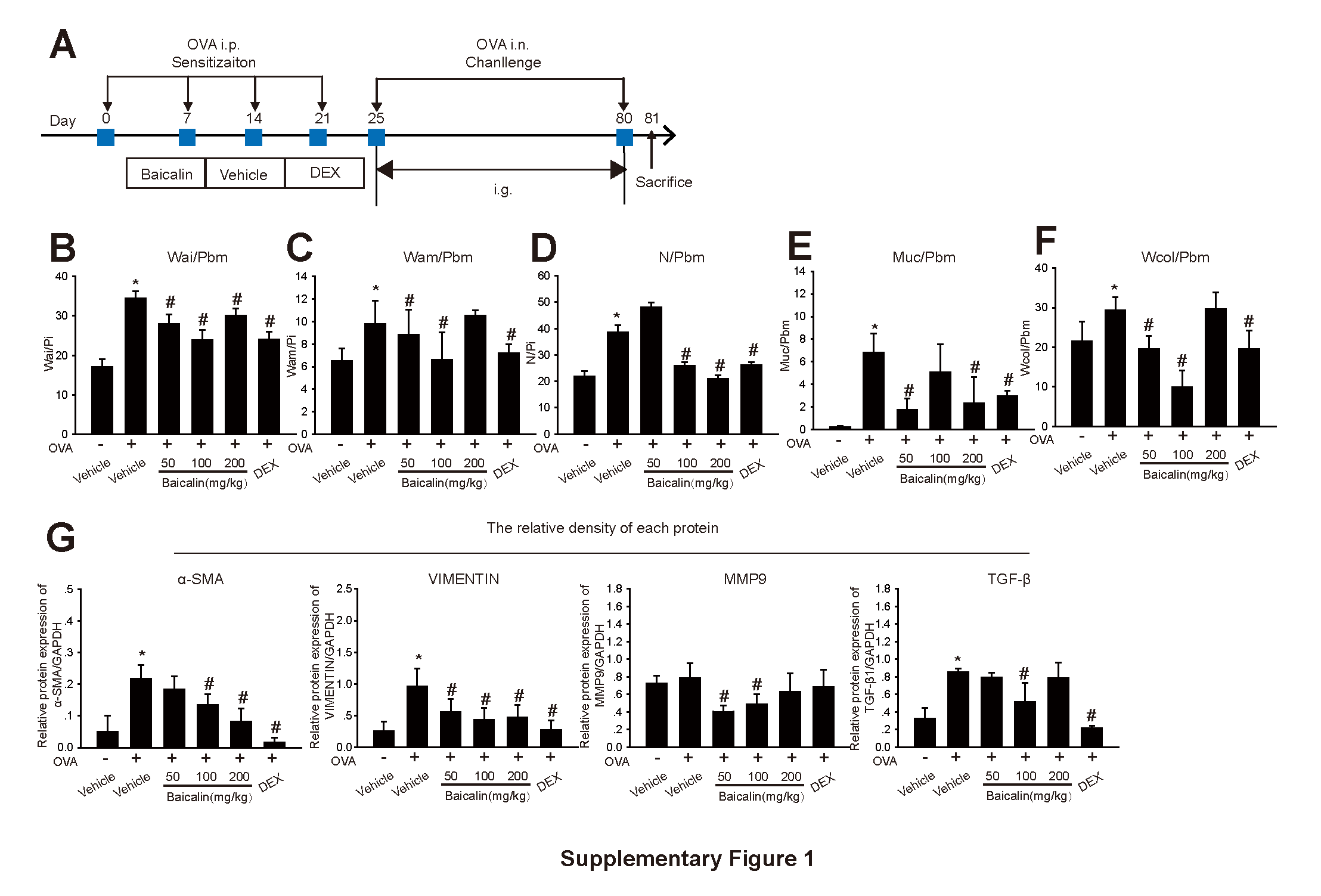


Supplementary figure 1: Effects of baicalin on airway remodeling indicators in mice. (A) Schematic diagram of the experimental protocol. (B) Airway remodeling-related indicators Wam/Pbm (B), Wai/Pbm (C), and N/Pbm (D) were measured from H&E staining. (E) Muc/Pbm was measured from PAS staining. (F) Wcol/Pbm was measured from Masson staining. (G) Relative density of α-SMA, VIMENTIN, MMP9, and TGF-β1in the lung. All data were shown as mean ± SEM. The data are representative of n ≥ 5 from each group. *^*^P* < 0.05 versus vehicle. *^#^P* < 0.05 versus OVA.


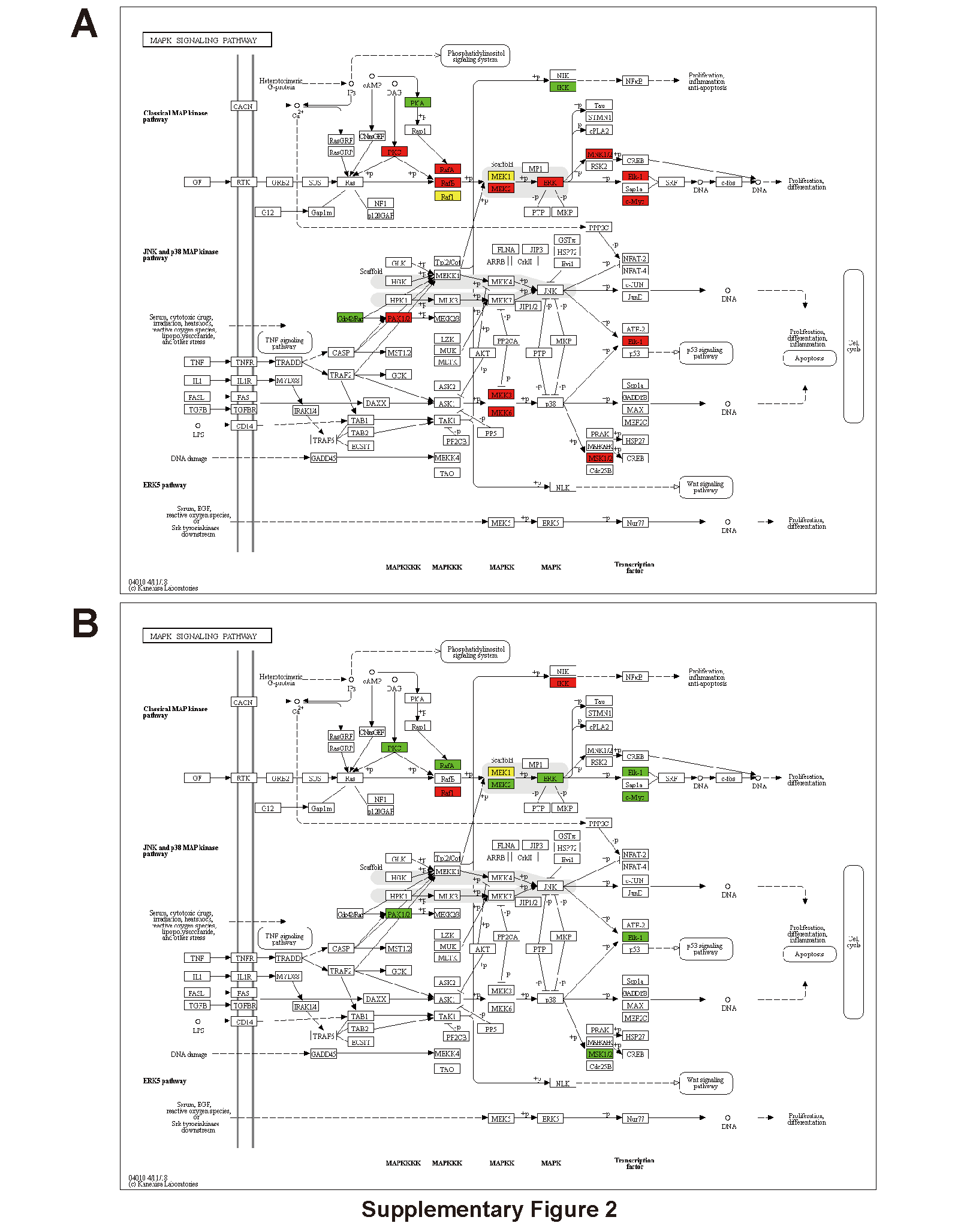


Supplementary figure 2: Differentially expressed proteins were screened out by an antibody array. (A) Differentially expressed proteins on the RAS signaling pathway between the CON and OVA groups. (B) Differentially expressed proteins on the RAS signaling pathway between the OVA and baicalin groups. All data were shown as mean ± SEM. The data are representative of n =5 mice from each group.
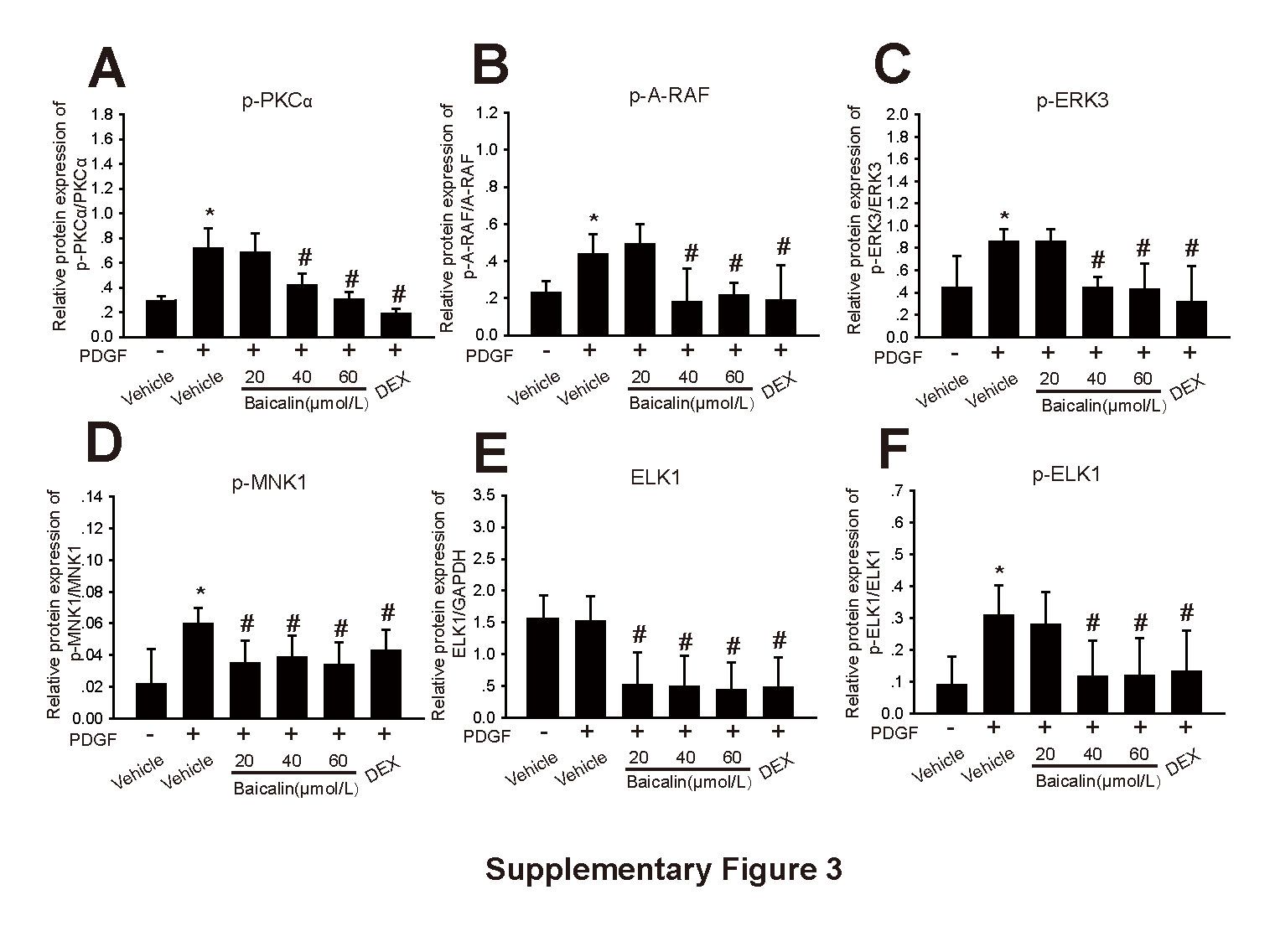


Supplementary figure 3: Baicalin inhibits the RAS signaling pathway activation. (A) p-PKCα, (B) p-A-RAF, (C) p-ERK3, (D) p-MNK1, (E) ELK1, and (F) p-ELK1 protein expression in ASMCs. All data were shown as mean ± SEM. The data are representative of n ≥ 6 from each group. *^*^P* < 0.05 versus vehicle. *^#^P* < 0.05 versus PDGF.
